# Moving toward a prevention strategy for osteoporosis by giving a voice to a silent disease

**DOI:** 10.1186/s40695-016-0016-0

**Published:** 2016-03-07

**Authors:** Karl J. Jepsen, Erin M. R. Bigelow, Melissa Ramcharan, Stephen H. Schlecht, Carrie A. Karvonen-Gutierrez

**Affiliations:** 1 Department of Orthopaedic Surgery, Biomedical Sciences Research Building, 109 Zina Pitcher Place, University of Michigan, Ann Arbor, MI 48109-2200 USA; 2 Department of Epidemiology, School of Public Health, University of Michigan, Ann Arbor, MI 48109-2029 USA

**Keywords:** Menopausal transition, Bone, Strength, BMD, Osteoporosis, Biomechanics

## Abstract

A major unmet challenge in developing preventative treatment programs for osteoporosis is that the optimal timing of treatment remains unknown. In this commentary we make the argument that the menopausal transition (MT) is a critical period in a woman’s life for bone health, and that efforts aimed at reducing fracture risk later in life may benefit greatly from strategies that treat women earlier with the intent of keeping bones strong as long as possible. Bone strength is an important parameter to monitor during the MT because engineering principles can be applied to differentiate those women that maintain bone strength from those women that lose bone strength and are in need of early treatment. It is critical to understand the underlying mechanistic causes for reduced strength to inform treatment strategies. Combining measures of strength with data on how bone structure changes during the MT may help differentiate whether a woman is losing strength because of excessive bone resorption, insufficient compensatory bone formation, trabeculae loss, or some combination of these factors. Each of these biomechanical mechanisms may require a different treatment strategy to keep bones strong. The technologies that enable physicians to differentially diagnose and treat women in a preventive manner, however, have lagged behind the development of prophylactic treatments for osteoporosis. To take advantage of these treatment options, advances in preventive treatment strategies for osteoporosis may require developing new technologies with imaging resolutions that match the pace by which bone changes during the MT and supplementing a woman's bone mineral density (BMD)-status with information from engineering-based analyses that reveal the structural and material changes responsible for the decline in bone strength during the menopausal transition.

The optimal management of osteoporosis remains unresolved [1]. Current diagnostic and treatment protocols for osteoporosis are initiated when an individual’s areal bone mineral density (aBMD) T-score falls below −2.5 [2]. Those with T-scores between −1 and −2.4 may also be treated depending on whether they have additional risk factors such as a prior fracture or a family history of fractures. With this treatment strategy, one has to become osteopenic or nearly osteoporotic to be diagnosed and treated for osteoporosis. Having to suffer a fragility fracture to be diagnosed and treated for osteoporosis is like having to suffer a heart attack to be diagnosed and treated for heart disease. Fortunately for cardiovascular medicine, adoption of pre-clinical measures such as blood pressure and serum lipoprotein profile and knowledge of major risk factors such as smoking status and obesity allow primary care physicians to treat individuals preventatively in advance of a catastrophic event. Regrettably for bone health, similar pre-clinical measures have not yet been well developed for osteoporosis. The current strategy generally identifies individuals with osteoporosis after they have lost an appreciable amount of bone and prophylactically treats them with the intent of restoring the BMD that was lost during the previous decade. This lagged diagnostic and treatment approach is particularly problematic for women as they uniquely experience the menopausal transition which is characterized by more rapid bone loss, thereby positioning them at greater fracture risk during the postmenopause. Further, this strategy is geared toward *restoration* rather than *prevention*, and consequently places women at risk of fracturing prior to treatment [2]. In this commentary, we emphasize the need for better prevention methods that include detection of changes in bone strength during the menopausal transition given knowledge of the significance of this life stage for bone health as women transition to old age. This strategy will give a voice to what has often been characterized as a silent disease. However, knowledge gaps remain regarding the ideal window for treatment and the type of information that would be clinically useful for a successful prevention strategy.

*Why is the menopausal transition important?* To transition the current strategy for treating osteoporosis from one that focuses on restoration toward one that focuses on prevention will require the identification of new biomarkers that monitor a woman’s bone health status earlier in life and that identify those women who are in need of early intervention. The menopausal transition is associated with rapid changes in serum levels of follicle-stimulating hormone (FSH), estradiol (E2), testosterone, and inhibin A and B as well as a rapid bone strength decline before the slower phase of postmenopausal bone loss begins [1, 3–8]. Longitudinal studies have shown that accelerated losses in BMD and bone strength begin ~1–2 years prior to the final menstrual period (FMP), continue at this rate until ~2–5 years after the FMP, and then slow appreciably thereafter [1, 6]. Women may lose as much as 33 % of their bone strength between 45 and 65 years of age before treatments for osteoporosis typically begin [9]. The timing and magnitude of bone mass and bone strength changes have been well described on a population average basis [1, 3–8, 10], but little is known about the individual-level differences in bone mass and bone strength during the menopausal transition.

*Why do details of bone loss matter?* Fracture prone sites like the wrist, spine, and hip are comprised of a relatively thin cortical shell that is supported by a highly organized mass of trabecular bone (Fig. 1). These skeletal structures resemble a bridge, which is comprised of a roadway (cortical bone) and its associated support structure (trabecular bone). Following normal wear and tear associated with daily usage, bits and pieces of the bridge will naturally begin to fail. Imagine one morning a sign is posted indicating there is a 5 % loss in the mass of the bridge and to proceed with caution. Would you drive over the bridge? You would likely want additional information to know whether the 5 % loss in bridge-mass included critical features like parts of the roadway or a supporting beam needed to hold up the road. Further, if you wanted to keep the bridge in good shape to avoid a catastrophic event, you would want to know which components were in need of repair and to set up a regular maintenance schedule. We propose that the same concepts for maintaining a bridge are needed to maintain bone strength.

**Fig. 1:**
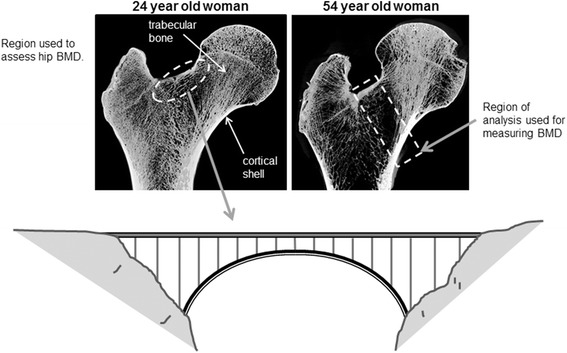
Sagittal sections derived from 3-dimensional nanoComputed Tomography images (nanotom-s; phoenix|x-ray, GE Sensing & Inspection Technologies, GmbH; Wunstorf, Germany) of cadaveric proximal femurs for two women, one 24 years old and the other 54 years old, convey how bone loss occurs non-uniformly with aging and predominantly in the region used to measure aBMD with DEXA. Maintaining bone strength is analogous to maintaining a bridge, where all critical structural components must be recognized and targeted to keep the structure intact and functioning to prevent a catastrophic failure event

*Which details of skeletal changes during the MT matter?* To date, risk prediction has focused mainly upon demographic characteristics, family history, medications, lifestyle characteristics (e.g., smoking, alcohol intake), fracture history, and aBMD [11]. aBMD has been used successfully for screening postmenopausal women who have osteoporosis, and identifying large changes in bone mass that may increase a woman’s risk of fracturing [2]. However, BMD is not sufficiently sensitive to monitor early declines in bone strength [9], which begin largely during the menopausal transition [1, 3]. It has been hypothesized that bone strength may be the more appropriate measure to predict fracture risk [9, 12, 13]. Bone strength is a measure of the amount of load or force it takes to cause a bone to fail, and thus is a critical parameter that differentiates whether a bone will break or not during a simple fall [12, 14]. However, current treatment paradigms often focus on maintaining BMD not strength. This distinction is important because a woman may lose bone mass without losing bone strength. This phenomenon exists because bone has adaptive features that allow it to compensate for bone loss to maintain strength [15]. Bone forming cells (osteoblasts) can deposit small amounts of tissue on the outer surface of the bone to mechanically offset the loss that occurs when the bone resorbing cells (osteoclasts) remove tissue on the inner surface [16–19]. Thus, bones tend to become wider with aging, a phenomenon that is thought to help maintain bone strength [16, 20, 21]. Because of the way bones are loaded during daily activities, only a small amount of new tissue needs to be deposited on the outer surface to offset a large amount of loss near the inner surface [21, 22]. Consequently, some women may show a net loss of bone mass while maintaining bone strength. Whether fracture risk depends on the degree to which the newly deposited tissue on the outer surface mechanically offsets the bone loss on the inner surface is not fully understood [20, 23]. Critically, the amount of new tissue deposited on the outer bone surface declines during the menopausal transition and is only a fraction of premenopausal levels by age 65 [23]. Thus, the window for most efficacious treatment for maintaining bone strength may be during the menopausal transition.

Knowing a woman’s bone strength is important, but is not sufficient by itself to inform clinicians on how best to treat women in a personalized preventative manner. The details of how bone changes with aging, in particular the balance between bone resorption and bone formation at all structural levels, are needed to inform clinicians how to keep bones strong during the menopausal transition and the postmenopausal years [21, 23]. Translational studies are needed to define the different ways in which women lose bone strength. Combining measures of strength with data on how bone structure changes during the MT may help differentiate whether a woman is losing strength because of excessive resorption, insufficient formation, or some combination of these factors. These outcomes may require different treatment strategies to maintain strength [24]. For example, women exhibiting excessive bone resorption may be better treated with an anti-resorptive therapy, whereas a woman with narrow bones and low periosteal expansion may be better treated with an anabolic therapy [25].

The armamentarium of prophylactic treatments for osteoporosis has matured faster than the clinical adoption of technologies that enable physicians to differentially diagnose and treat individuals in a preventive manner. Serum biomarkers of bone resorption and formation are informative of system-wide changes in bone structure, but these biomarkers do not inform on the magnitude and location of bone loss and gain at individual anatomical sites (e.g., femur, distal radius, spine) [26], which is needed to understand how bone strength changed. Periosteal expansion, endocortical resorption, trabecular loss, and increased porosity can all be measured using existing technologies [23, 27–31]. Hand radiographs offer an inexpensive alternative from which bone structural changes can be assessed over time [32–36], possibly beginning at a premenopausal age [22]. The potential costs and apparent impracticality of acquiring structural assessments at multiple time points are recognized deterrents to general adoption of a prevention strategy. However, additional translational research that focuses on understanding how precipitating biological events occurring during the menopausal transition define fracture risk later during the aging process would break the silence of this disease, and potentially identify new technologies, biomarkers, or baseline characteristics that accurately predict individual trajectories of bone loss and gain over time and that can be incorporated into the clinic inexpensively and conveniently.

## Conclusions

Accumulating evidence indicates that there are substantive changes in bone strength during the menopausal transition. Many challenges remain to give a voice to a silent disease by better understanding how events during the menopausal transition affect fracture risk during the postmenopausal years. These include identifying biomarkers that reveal the details of bone loss and gain; identifying individual or combinations of biomarkers that can be used to identify women in need of early intervention; testing whether peripheral skeletal sites (e.g., metacarpals, tibia, wrist) can be used as proxies of strength declines at fracture-prone sites like the proximal femur and spine; understanding how changes in hormones affect the balance between bone resorption and formation at all structural levels and how this balance affects strength; and conducting translational studies that identify the mechanisms responsible for inter-individual differences in skeletal aging, particularly during the menopausal transition. Taking advantage of the data within existing longitudinal studies of women transitioning through the menopause, including the Michigan Bone Health and Metabolism Study [37], the Study of Women’s Health Across the Nation [8, 38], and the Melbourne Women’s Midlife Health Project [39], will be extremely helpful in resolving these knowledge gaps and moving from a restoration to a prevention treatment strategy.
